# Fat dads must not be blamed for their children's health problems

**DOI:** 10.1186/1741-7015-11-30

**Published:** 2013-02-06

**Authors:** Gudrun E Moore, Philip Stanier

**Affiliations:** 1 Clinical and Molecular Genetics Unit, UCL Institute of Child Health, 30 Guilford Street, London, WC1N 1EH, UK; 2 Neural Development Unit, UCL Institute of Child Health, 30 Guilford Street, London, WC1N 1EH, UK

**Keywords:** Insulin-like growth factor 2, paternal obesity, DNA methylation, genomic imprinting

## Abstract

The relationship between the parental genomes in terms of the future growth and development of their offspring is not critical. For the majority of the genome the tissue-specific gene expression and epigenetic status is shared between the parents equally, with both alleles contributing without parental bias. For a very small number of genes the rules change and control of expression is restricted to a specific, parentally derived allele, a phenomenon known as genomic imprinting. The insulin-like growth factor 2 (*Igf2/IGF2*) is a robustly imprinted gene, important for fetal growth in both mice and humans. *In utero IGF2 *exhibits paternal expression, which is controlled by several mechanisms, including the maternally expressing untranslated *H19 *gene. In the study by Soubry *et al.*, a correlation is drawn between the *IGF2 *methylation status in fetal cord blood leucocytes, and the obesity status of the father from whom the active *IGF2 *allele is derived through his sperm. These data imply that paternal obesity affects the normal *IGF2 *methylation in the sperm and this in turn alters the expression of *IGF2 *in the baby.

## Background

The "parental conflict hypothesis" is the most widely accepted explanation for genomic imprinting [1]. This hypothesis is based on the conflicting interests of the maternal and paternal genomes during the offspring's growth and development. The paternal genes are directed towards the growth and fitness of the fetus, enhancing the chances of his genome to be passed on to successive generations. The maternal genome attempts to limit fetal growth in order to distribute equal resources to each member of her litter, while ensuring her own survival during birthing to be able to reproduce again. Studies of imprinted genes generally support this model, with one of the most striking examples being the mouse insulin-like growth factor II (*Igf2) *[2,3] and its chelating insulin-like growth factor II receptor (*Igf2r)*, which show reciprocal imprinting effects and associated growth patterns [4]. *IGF2 *is an important human fetal growth promoter that is regulated by the nearby *H19 *gene, which is also oppositely imprinted in humans [5,6]. For a handful of these imprinted, haploinsufficient and fetally expressing genes, including *IGF2*, their transcription is potentially more vulnerable. Alterations in their regulation through either mutation or disturbed epigenetic processes may lead to functional abnormalities and disease states.

### Methylation variation and the link to functional expression

Good examples of epigenetic syndromes are the rare imprinted human growth disorders Beckwith-Wiedemann syndrome (BWS) and Silver-Russell syndrome (SRS), where *IGF2 *and *H19 *methylation are mechanistically implicated and are considered to be functionally important [7]. It now seems evident that disruption to epigenetic marks, such as DNA methylation or histone modifications, can affect the structure of chromatin and, therefore, the binding of transcription factors and other regulatory proteins [8]. DNA methylation is relatively easy to study from stored DNA samples and generally involves making comparative assessment of the methylation variation at CpG islands, promoters or differentially methylated regions (DMRs) between control and experimental groups of subjects. Importantly, there are both tissue- and age-specific methylation profiles and for many such marks, there is a high level of intra- and inter-individual variation that appears to have no phenotypic consequence. Caution must, therefore, be employed when analyzing these data. It is clear that more detailed and carefully controlled studies will be necessary to gain a complete understanding of key methylated marks and their variation relevance to development and disease [9].

### *IGF2 *expression in fetal samples and links to growth

There have been many studies investigating the effect of human placental expression of *IGF2 *and its receptors on fetal growth. Given the limitations of this type of study it is not surprising that they are usually confined to samples obtained at the time of birth. Unfortunately, many of these studies seem to report conflicting results. For example, in one study no correlation was reported between *IGF2 *expression and birth weight [10], whereas others have shown that in small for gestational age (SGA) babies' pregnancies, *IGF2 *expression is either increased [11], or decreased [12], including at the protein level [13]. Nevertheless, it seems likely that IGF2 should play an important role in dictating fetal growth, even though this effect may not be reliably detected in term placenta.

### This study and future research

In the study by Soubry *et al*. [14], the authors assess the relationship between the physiological body mass index (BMI) or obesity status of the parents with the level of methylation seen for the *IGF2 *and *H19 *gene DMRs in the leucocytes from cord blood of their newborns (n = 79). They find an association between the father's obesity status and the loss of methylation at the DMR linked to *IGF2 *but not the *H19 *DMR. The main caveats of this study are the small numbers and the mixture of ethnicities. In addition, it has not been shown in this study whether a small change in methylation (in this case, 4 to 5%) at a DMR has any truly functional significance at the phenotype level. The authors point to their previous finding that a 5% loss of methylation could lead to a 10% increase in serum concentration of IGF2 [15]. While it seems feasible that this might have both short and longer term consequences for health, it has not as yet been associated with an actual change to fetal growth. The Soubry *et al*. [14] study, for example, found no correlation between methylation or parental obesity and birth weight. Nevertheless, this study provides the first evidence that hypo-methylation of the regulatory control region for *IGF2 *is influenced by increased paternal BMI and the study makes a compelling case for a larger analysis to be performed. It is surprising and counter intuitive to note that a similar effect of hypomethylation on the *IGF2 *DMR is seen both for paternal obesity and maternal preconceptual famine [16]. One might assume that the father's BMI was also affected by the famine.

## Conclusions

Fetal growth and development *in utero *is complex and will involve multiple gene pathways, many of which will not be haploinsufficient or driven by one parental allele. It is important and of particular human interest to allude to the differing roles of the parents in the developmental outcome of the future offspring. Without even considering imprinted genes, it is obvious that the two parents must have different physiological roles in pregnancy. We must consider the whole genome/epigenome/metabolome in an unbiased way, involving all key genes and networks. It is also important to critically assess environmental players. It is tempting to over-emphasize the role of a small number of parent-of-origin expressing genes and to speculate about the effects of modest variation in methylation, but we must not be too hasty to blame either parent for their offspring's health outcomes without being certain that these effects are consequentially robust.

## Abbreviations

BMI: Body Mass Index; BWS: Beckwith-Wiedemann syndrome; DMR: Differentially methylated region; Igf2r: Insulin-like growth factor-2 receptor; Igf2/IGF2: Mouse/Human Insulin-like growth factor-2; SGA: Small for Gestational Age; SRS: Silver-Russell syndrome

## Competing interests

The authors declare that they have no competing interests.

## Authors' contributions

GEM and PS both contributed to drafting and editing of the manuscript and both read and approved the final manuscript.

## Authors' information

GEM and PS have a specialist interest in human fetal growth and development with a view to understanding pregnancy complications. GEM was awarded an Honorary Fellowship of the Royal College of Paediatrics and Child Health in 2012 and is the Co-Director the Baby Bio Bank (http://www.ucl.ac.uk/babybiobank).

## Pre-publication history

The pre-publication history for this paper can be accessed here:

http://www.biomedcentral.com/1741-7015/11/30/prepub
